# Viscoelastic Properties of Differentiating Blood Cells Are Fate- and Function-Dependent

**DOI:** 10.1371/journal.pone.0045237

**Published:** 2012-09-27

**Authors:** Andrew E. Ekpenyong, Graeme Whyte, Kevin Chalut, Stefano Pagliara, Franziska Lautenschläger, Christine Fiddler, Stephan Paschke, Ulrich F. Keyser, Edwin R. Chilvers, Jochen Guck

**Affiliations:** 1 Cavendish Laboratory, Department of Physics, University of Cambridge, Cambridge, United Kingdom; 2 Department of Medicine, University of Cambridge School of Clinical Medicine, Addenbrooke's and Papworth Hospitals, Cambridge, United Kingdom; 3 Department of Surgery, University of Ulm, Ulm, Germany; 4 Biotechnology Center, Technische Universität Dresden, Dresden, Germany; Emory University/Georgia Insititute of Technology, United States of America

## Abstract

Although cellular mechanical properties are known to alter during stem cell differentiation, understanding of the functional relevance of such alterations is incomplete. Here, we show that during the course of differentiation of human myeloid precursor cells into three different lineages, the cells alter their viscoelastic properties, measured using an optical stretcher, to suit their ultimate fate and function. Myeloid cells circulating in blood have to be advected through constrictions in blood vessels, engendering the need for compliance at short time-scales (<seconds). Intriguingly, only the two circulating myeloid cell types have increased short time scale compliance and flow better through microfluidic constrictions. Moreover, all three differentiated cell types reduce their steady-state viscosity by more than 50% and show over 140% relative increase in their ability to migrate through tissue-like pores at long time-scales (>minutes), compared to undifferentiated cells. These findings suggest that reduction in steady-state viscosity is a physiological adaptation for enhanced migration through tissues. Our results indicate that the material properties of cells define their function, can be used as a cell differentiation marker and could serve as target for novel therapies.

## Introduction

Biological cells are viscoelastic materials with a wide range of adaptable mechanical properties [1], [2]. Cellular mechanical properties are known to alter in disease [3] and during physiological processes such as differentiation [4], motility [5], cell cycle [6] and apoptosis [7]. However, understanding of the functional relevance of such alterations in cell mechanics is incomplete. As stem cells differentiate into specialized cells, not only are their mechanical properties modulated but many of their biochemical characteristics are altered as well. Thus, common methods for monitoring differentiation take advantage of the biochemical changes involving gene expression and protein synthesis [8]. Such methods include RNA microarrays, reverse transcription polymerase chain reactions, blotting techniques and immunofluorescent labeling in flow cytometry and microscopy [9]. Despite their success in unraveling some of the molecular pathways involved in differentiation, these methods are not ideally suited because they all rely on external markers, such as fluorescent antibodies, potentially causing unwanted signaling and altering cell properties, or they are destructive. Moreover, cells are seen as mere loci where biochemistry happens, largely ignoring that they are actually material objects having to navigate a 3D physical environment.

The material properties of cells have thus recently moved into the focus of characterization. Changes in the mechanical properties of stem cells as they differentiate have been reported and most results show an increase in stiffness with differentiation, congruent with a build-up of the cells' cytoskeleton [4], [10], [11]. However, there are some cell types that do not become stiffer with differentiation. For instance, differentiated neutrophils are more compliant than their undifferentiated hematopoietic precursors [5]. These contrary trends pose the obvious question, whether there is a general underlying principle in the evolution of material properties of cells during differentiation?

To address this question, we tracked mechanical changes when the well-established model human myeloid precursor cells, HL60 [12], were differentiated *in vitro* into three mature lineages: neutrophils, monocytes and macrophages. *In vivo*, all three lineages ultimately originate from the same hematopoietic stem cells (HSCs) residing in the bone marrow [13]. There, HSCs undergo self-renewal and differentiation, giving rise to red blood cells, white blood cells and platelets, which are the cellular constituents of blood [13]. White blood cells, or leukocytes, are part of the immune system, which protects and defends the body against infections and the spread of tumors. Neutrophils, monocytes and macrophages are the main leukocytes that collaborate in destroying invading pathogens. In a normal adult, over 5–10×10^10^ neutrophils are produced in the bone marrow daily [14]. These circulate through the blood for a few hours until activated by chemical signals to extravasate into tissues where they carry out crucial antimicrobial activities at sites of infections including the phagocytosis of pathogens. Monocytes also circulate through blood vessels and extravasate into tissues in response to infection. Once they enter tissues, monocytes become macrophages [15]. The macrophages are phagocytes present in almost all human tissues, where they engulf and digest pathogens and dead cells. Unlike neutrophils, which can survive in tissues for only a few days, macrophages can survive for months and are therefore called resident macrophages. Thus, neutrophils, monocytes and macrophages have to deal with different physical challenges in their microenvironments and probably have to adjust their mechanical properties accordingly.

Here we show that all three mature lineages can be distinguished from the undifferentiated cells and from one another based on a single material property, the creep compliance. We further demonstrate that the ensuing material properties are actually tailored towards the ultimate function of the cells. Moreover, we find the same, but larger characteristic differences in compliance between primary human HSCs (CD34+ cells), primary human neutrophils and monocytes, confirming the physiological importance of the findings from the cell line model.

## Results

### Compliance of myeloid cells is lineage-specific

**Figure 1:**
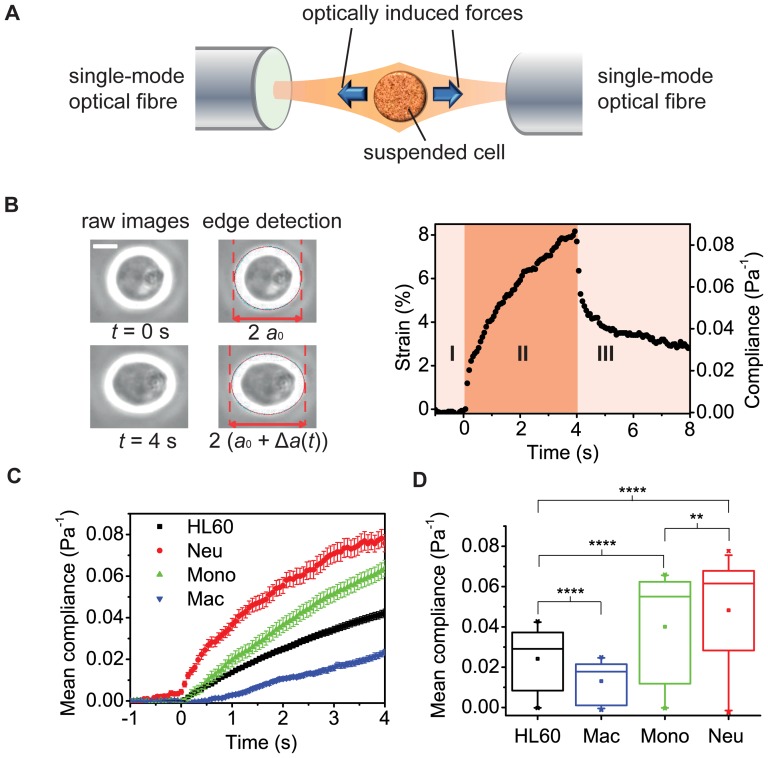
Creep compliance test distinguishes between cell lineages following differentiation. **A**, Schematic of the optical stretcher (OS), showing two diverging, counter-propagating laser beams emanating from single-mode optical fibres. **B**, A cell is held at trapping stress of 1.7±0.1 Pa at time *t* = 0 s. The trapped cell is stretched when the optical stress is increased to 6.0±0.1 Pa, producing an axial strain of 8.17±0.05% at *t* = 4 s, for the cell shown. The graph shows both the strain and compliance profiles for this one cell, illustrating the single cell resolution of the OS method. Scale bar is 10 µm. **C**, Representative compliance profiles for all three fully differentiated lineages and for the undifferentiated cells (*n* = 89). For neutrophils (Neu; *n* = 75) and monocytes (Mono; *n* = 85), the compliance increased, while for macrophages (Mac; *n* = 52) the compliance decreased. **D**, Box plots of the average compliance during the four seconds of stretching. For all three differentiated lineages, the entire compliance profile, as well as the peak compliance at 4 s (not shown), were significantly different from undifferentiated HL60 cells, where **** and ** indicate significant differences with *p* values of <0.0001 and <0.01, respectively.

To measure the compliance of cells that are normally in suspension in their physiological environment, a method that preserves this suspended state is required. Here, we used a microfluidic optical stretcher (OS), which is a specific dual-beam trap, capable of inducing well-defined stresses on whole single cells in suspension and therefore useful for measuring overall cell deformability and creep compliance [16], [17]. For details, see the Methods section. Briefly, the OS consists of two diverging, counter-propagating Gaussian laser beams, which emanate from single-mode optical fibres (Fig. 1A). During a typical experiment, cells are introduced into a microfluidic delivery system, serially trapped and then stretched along the laser beam axis (Fig. 1B). The time-dependent strain is extracted from the video camera images, normalized by the peak stress applied and a geometric factor [18], to obtain the creep compliance for each cell. For the cell shown, the peak compliance (at *t* = 4 s) is 0.083±0.005 Pa^−1^.

The precursor (HL60) cells are induced to differentiate using standard protocols (see Methods and Methods S1) and then repeatedly measured with the microfluidic OS over the course of several days to track the evolution of their viscoelastic properties. Mature neutrophil- and monocyte-like cells are obtained from HL60 cells 72–96 hours after induction of differentiation. Macrophage-like cells are obtained from 24 hours after induction of differentiation. Our results show that a creep compliance measurement distinguishes between these three cell lineages following differentiation to mature cells (Fig. 1C, D). For all three lineages, the peak compliance at *t* = 4 s of stretching and the entire compliance profile are significantly different from undifferentiated cells (*p*<0.0001). Moreover, even though both neutrophils and monocytes become more compliant, we still find significant differences between both their peak compliances and their entire compliance profiles (*p*<0.01). These lineage-specific differences in compliance are reproducible at higher stresses and in different OS set-ups with varying geometries (Fig. S2). Of note, concomitant changes in cell size during differentiation (Fig. S3) are explicitly considered in the geometric factor *F_g_*, which is used for the proper normalization of the compliance. Thus, the creep compliance is a true material property of these cells and can be used as sensitive yet robust, non-invasive and inherent physical marker to monitor differentiation and even lineage specification.

### Lineage-specificity is reflected in detailed viscoelastic properties

**Figure 2:**
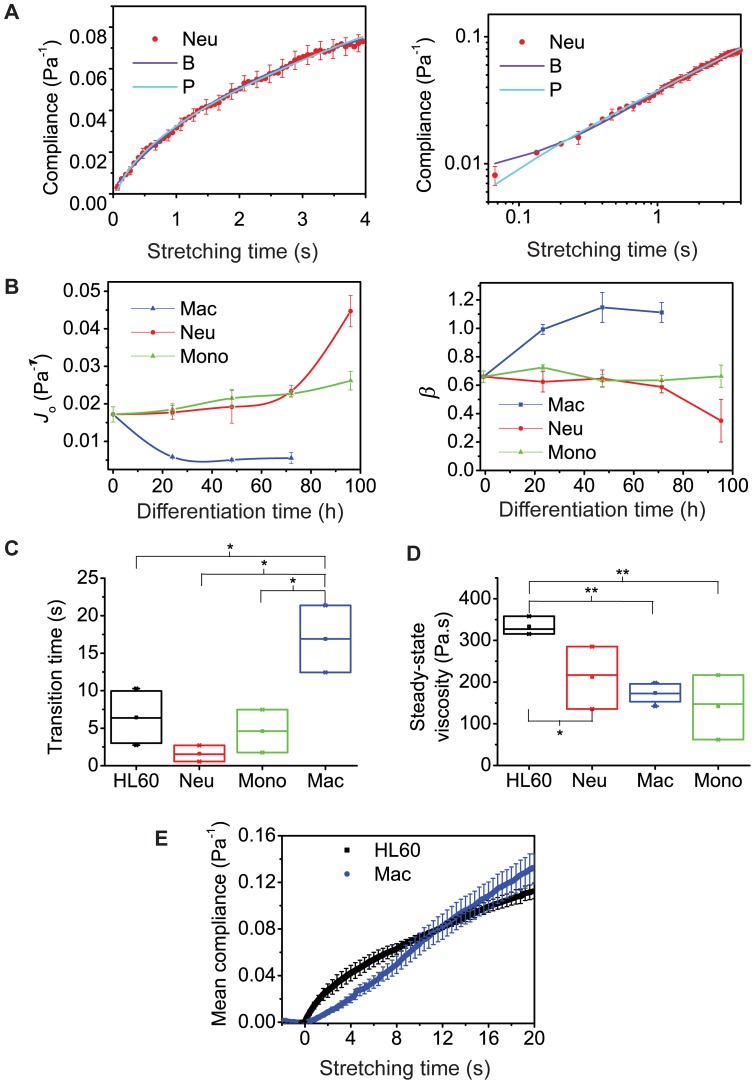
Power law and mechanical models describe fate-specific viscoelastic properties of cells during the course of differentiation. **A**, Power law fit (P) and one of the mechanical models, here Burgers' model fit, (B) used to extract viscoelastic parameters. For time-scales above 0.1 s and much longer, both power law and mechanical models offer overlapping insights. **B**, Evolution of initial compliance, *J_o_*, and power law exponent, *β*, during the differentiation of cells over several days. *J_o_* characterizes the lineage-specific changes in compliance as cells become fully differentiated at 24 h (macrophages) and 96 h (monocytes and neutrophils) after induction of differentiation. The power law exponent *β* is about 1 for macrophages, indicating liquid-like behavior. The exponent decreases for neutrophils showing increased solid-like behaviour as cells become fully differentiated at 96 h, but stays constant for monocytes, apparently consistent with their state as immature macrophages. **C**, Time-scales, at which cells change their viscoelastic behaviour, defined as the ratio *η*_2_/*E*_2_ from the transient part of viscoelastic models. **D**, All differentiated lineages have a lower steady-state viscosity. **E**, Macrophages are less compliant than undifferentiated HL60 cells on short time creep (4 s) and more compliant on longer creep test (20 s). The * and ** represent significant differences with *p* values<0.05 and <0.01, respectively.

To gain new insights into cell biological function using cell material properties further analysis of the compliance results is required. We employed both basic mechanical and power law models to extract detailed viscoelastic parameters (Fig. 2A; see also Fig. S4 and Methods S1). The equation for the power law model that fitted the data best required an offset *A*,
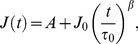
(1)where *J*(*t*) is the time-dependent compliance, *J*_o_ is the initial compliance, τ_0_ = 1 s and *β* is the power law exponent. The lineage specific modulation of compliance found in the raw compliance curves (Fig. 1C) is manifested in the power law fits in the initial compliance, *J*_o_. Tracking both parameters over the course of several days (Fig. 2B), this parameter *J*_o_ confirms that all macrophage-like cells (24–72 h after induction of differentiation) are significantly less compliant than undifferentiated cells while neutrophil- and monocyte-like cells become significantly more compliant (*p*<0.0001). Moreover, neutrophils are more compliant than monocytes at 96 h (*p*<0.0001). In the power law model, a purely elastic solid has a power law exponent of 0 and a purely viscous fluid has a power law exponent of 1. For macrophages the exponent *β* increases from 0.65 to about 1 within the first 24 h showing increasing fluid-like behaviour, while it decreases for neutrophils to 0.35 right before they become fully differentiated at 96 h showing progressively solid-like behaviour compared to the undifferentiated cells (Fig. 2B). The exponent *β* is nearly constant for monocytes, distinguishing them from neutrophils.

Since power law models assume time scale independence, which may not generally hold for biological cells, we also used specific viscoelastic models for fitting to the compliance data. The best-fitting viscoelastic model for undifferentiated HL60 cells, and differentiated neutrophils and monocytes was the so-called Burgers' model (B),
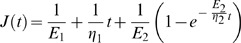
(2)while the best-fitting model for macrophages was the standard linear liquid model,
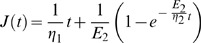
(3)where *E*_1_ describes the instantaneous elastic response found in all cell types except macrophages. The mere fact that macrophages are mechanically best represented by a liquid model, while the other cells have a more solid character, further emphasizes the emerging differences. The last term in equations (2) and (3) determines the transient response, which was found in all cell types. Clearly, it is possible to obtain the steady-state viscosity, *η*_1_, when the transient response becomes constant. For comparisons, we focused on the fit parameters found in all cell types, namely, the transient and steady-state parameters, and found the characteristic time-scales, *τ*, at which cells change their viscoelastic behaviour (Fig. 2C and Fig. 2D, respectively). Here, *τ* is given by the ratio of transient viscosity, *η*_2_, and elasticity, *E*_2_. The undifferentiated cells had a transition time of *τ*≈7 s. For monocytes and neutrophils, *τ* was 5 s but not significantly different from undifferentiated cells. For macrophages it was over 15 s, and was different from all other cell types (*p*<0.05) reflecting a reduced elasticity, *E*_2_. Regarding the steady-state viscosity, *η*_1_, all three differentiated cell types showed over 50% reduction compared to the precursor cells (Fig. 2D). For macrophages, *η*_1_ = 174.0±10.2 Pa s, while neutrophils and monocytes had *η*_1_ = 212.5±43.3 Pa s and 142.1±44.7 Pa s, respectively. It is intriguing that we cannot obtain *η*_1_ for undifferentiated cells within the 4 s of stretching but can do so for the differentiated cells. Extrapolating the viscoelastic model up to 20 s predicts a steady-state viscosity of over 300 Pa s for the undifferentiated cells. To obtain their steady-state viscosity directly within the actual data range, we carried out 20 s creep tests for the undifferentiated cells (*N* = 3 experiments) and found *η*_1_ = 333.5±26 Pa s, which is more than 50% higher than the values for all three differentiated cell types. The lower *η*_1_ of all differentiated cells implies that beyond some transient regime, all three differentiated cells should be more compliant than undifferentiated cells. We therefore validated this implication by also performing 20 s creep tests for macrophages, which are less compliant than undifferentiated cells at the 4 s creep testing. Indeed, the macrophages are significantly more compliant at 20 s creep testing (Fig. 2E). Succinctly, monocytes and neutrophils are elastically compliant cells at short time-scales. The lower steady-state viscosity is an apparent indication of their ‘latent’ fluid-like behavior at long time-scales. Macrophages, on the other hand, are generally more fluid-like cells and are less compliant than undifferentiated cells at short time-scales but more compliant at long time-scales.

### Material properties of cells are function-dependent

**Figure 3:**
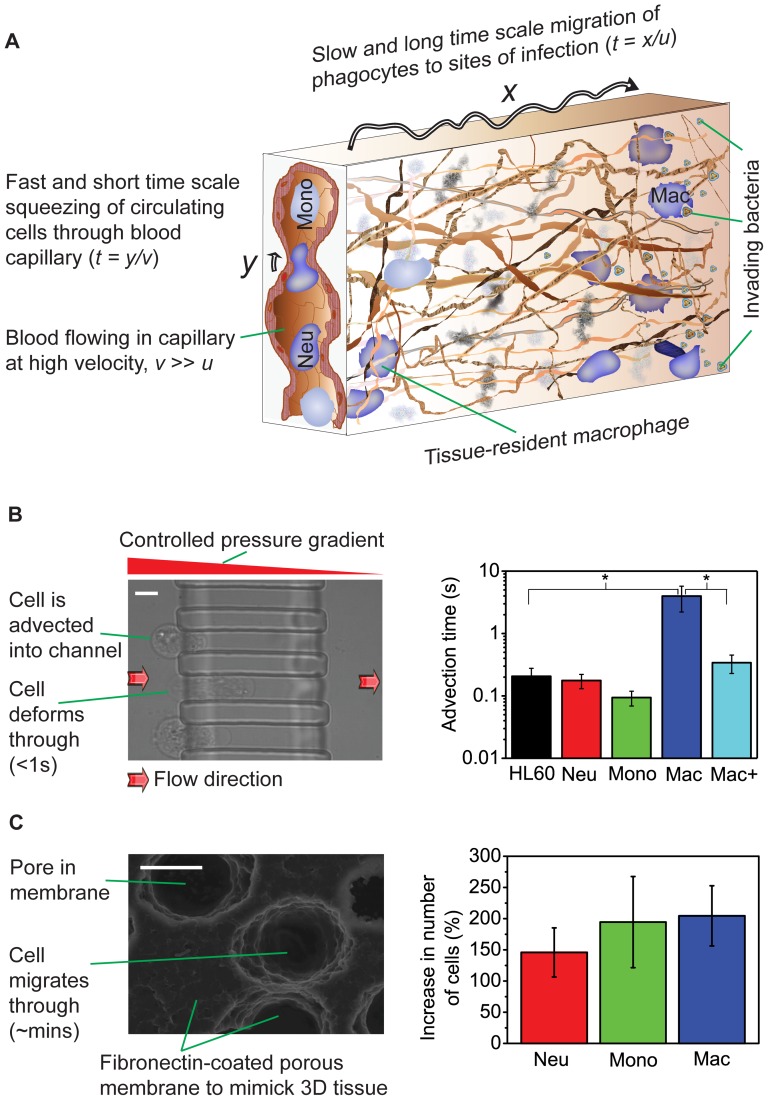
Time-scales and niche-specific relevance of cell viscoelasticity. **A**, *In vivo*, neutrophils and monocytes navigate constrictions smaller than themselves (*y*) and deform rapidly as illustrated (<1 s). Macrophages on the other hand, as well as neutrophils and monocytes after extravasation, migrate through tissue, which occurs over much longer time-scales. **B**, Short time scale advection of cells through the 12 µm×12 µm channel at a pressure of 20 mbar. Macrophages (*n* = 50) require an average advection time (from entry to exit) of 3.98±1.77 s, an order of magnitude longer than all other cell types and with statistically significant difference (*p*<0.05). There are no statistically significant differences between the advection times of HL60 (*n* = 24), neutrophils (*n* = 36), monocytes (*n* = 50) and macrophages treated with 2 µM Cytochalasin D shown as Mac+ (*n* = 67). Scale bar is 10 µm. **C**, Long time scale migration of the three mature cell types and precursors in Boyden chamber assays, where cells squeeze actively through a thick, porous membrane. After 3 h, significantly (*p*<0.0001) more of all three differentiated cell types have migrated than the undifferentiated, more viscous cells. Scale bar is 10 µm.

**Figure 4:**
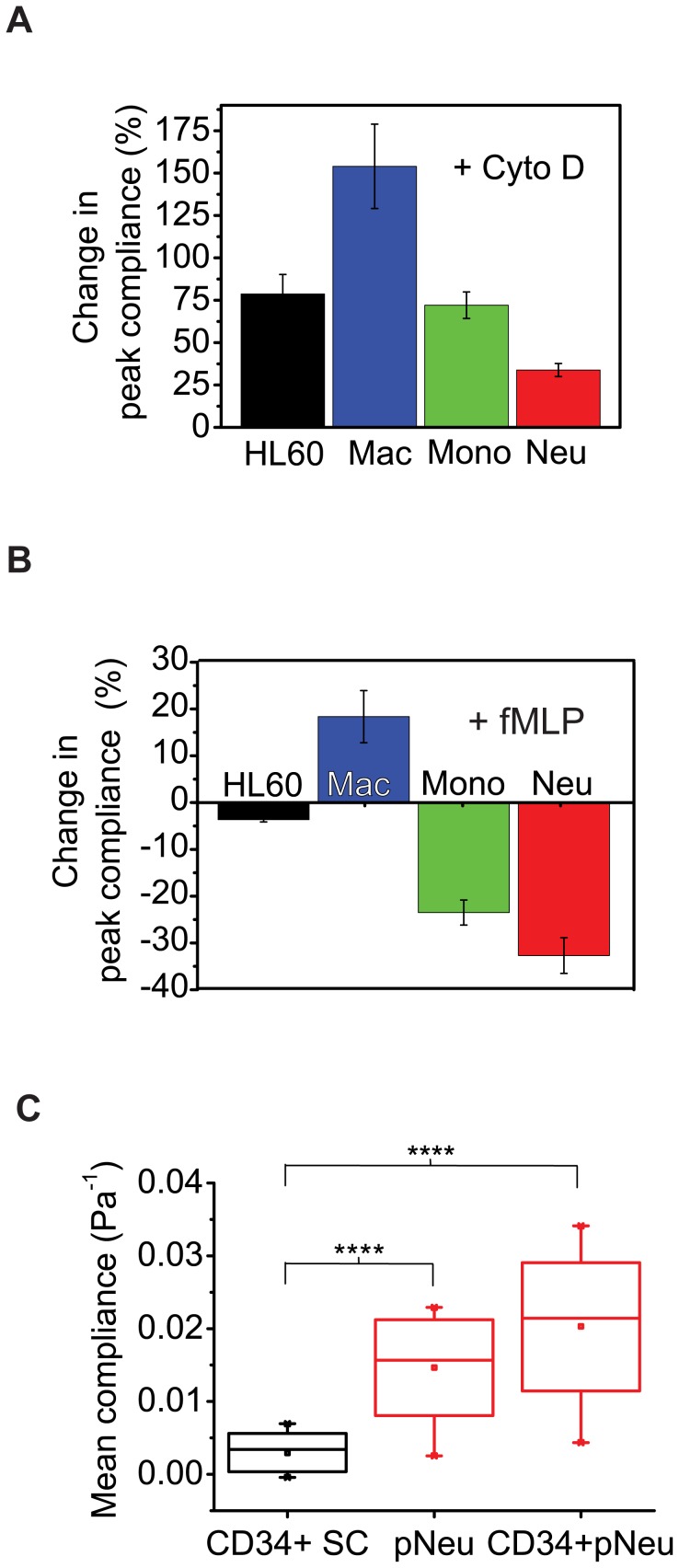
Pharmacological and functional assays and primary cell results. **A**, The cytoskeletal drug cytochalasin D (cyto D) disrupts filamentous actin, leading to increase in compliance (over 40%) due to actin depolymerization in all cell types. **B**, As a functional assay using the chemoattractant fMLP, all differentiated cell types responded to fMLP in a manner consistent with their *in vivo* counterparts. **C**, Primary human neutrophils and CD34+-derived neutrophils are respectively about 3-fold and 4-fold more compliant than CD34+ primary human cord blood stem cells. The **** indicates a significant difference with a *p* value of <0.0001.

These distinct rheological properties, robustly emerging towards the respective end of the differentiation into the mature phenotypes, suggest an intriguing hypothesis, where the material properties might be physical prerequisites for specific functions of the mature cell types. After all, circulating neutrophils and monocytes have to repeatedly squeeze through microvasculature constrictions smaller than their diameter while being advected along at high speed [19] (Fig. 3Aa). Since these are rapid impact processes, the cells need to be elastically compliant at short time-scales (<1 s), which the macrophages are not. To test this prediction we used pressures in the physiological range (5–20 mbar) to send cells through channels in PDMS with inner cross-sections of 10 µm×12 µm and 12 µm×12 µm, fabricated by photolithography [20] using PDMS (see Methods S1). To pass through the 12 µm×12 µm channel at a pressure of 20 mbar, macrophages required an average advection time (from entry to exit) of 3.98±1.77 s, an order of magnitude longer than all other cell types (Fig. 3B; *p*<0.05). At this pressure, macrophages could hardly be advected at all through the narrower 10 µm×12 µm channel (see Video S1) although all other cell types went through (see Video S2) even at a lower pressure of 10 mbar (Fig. S5). However, macrophages treated with 2 µM cytochalasin D, which depolymerizes filamentous actin [21], were 40% more compliant (Fig. 4A) and had an average advection time of 0.34±0.11 s, which is not significantly different from those of neutrophils, monocytes and precursors (Fig. 3B). These results confirm that cell compliance is the major limiting factor controlling the rapid passage of cells through narrow constrictions at short time-scales.

As phagocytes, all three mature cell types respond to microbial infection and so have to migrate to and through tissues to sites of infection. The crowded environment of tissues, as well as the absence of fluid pressure as the driving force (as in blood), makes tissue migration a process occurring at much longer time-scales (Fig. 3A). To test whether the cells' material properties are also in line with this second cell function, we compared the migratory ability of the three mature cell types with that of their precursors in Boyden chamber assays [5], where cells have to squeeze actively through a thick, porous membrane (Fig. 3C and Methods S1). After 3 h, more cells of all three differentiated lineages had migrated through the pores than the undifferentiated cells. Of interest, macrophages and monocytes (which ultimately become macrophages *in vivo*) both showed a higher relative increase in migration ability of 204% and 194%, respectively, compared to 145% for neutrophils (Fig. 3C). It is quite remarkable that an *in vitro* migration assay, which mimics *in vivo* migration in tissue over long times, correlates with the lowered steady-state viscosity of differentiated myeloid cells. The obvious interpretation is that the lowered steady-state viscosity is a physical prerequisite for ‘flowing’ through tissues over long time-scales (Fig. 3A). The fact that macrophages do not retain the high compliance of monocytes at short time-scales suggests an adaptation of their mechanical property to their cellular niche: they are tissue-resident cells and squeezing through tissue is a long-time process which does not require short-time compliance. These *in vivo* exigencies appear to explain our results, engendering insights into cell circulation and migration behaviour. In fact, recent *in vitro* studies show that matrix structural configuration determines 3D migration modes of human macrophages [22]. Our results therefore suggest novel cell mechanical-property-based therapeutic targets for blood/immune system dysfunctions where aberrant circulatory and migratory functions of neutrophils and monocytes/macrophages are implicated, such as sepsis [23], artherosclerosis [15] and chronic obstructive pulmonary disease [24].

### Confirmation of results with functional assays and primary cells

To ascertain the biological relevance of our results we tested whether the compliance measured is dictated by the cytoskeleton of cells, by disrupting filamentous actin using cytochalasin D. F-actin is a major determinant of cellular mechanical properties *in vitro* and *in vivo* [25]. Indeed, in all cell types we found over 40% increase in compliance due to actin depolymerization (Fig. 4A). Also the transition time dropped from 5 s or 15 s to less than 0.8 s in all cell types (Fig. S6).

Furthermore, we carried out myeloid functional assays on the differentiated cells using the chemo-attractant *N*-formyl-methionine-leucine-phenylalanine (fMLP). *In vivo* monocytes, neutrophils and macrophages express membrane receptors for bacterial fMLP which allow them to respond to chemotactic gradients, while fMLP-mediated chemotaxis is not detectable with undifferentiated HL60 cells [26]. Indeed, all three differentiated cells types (but not the undifferentiated cells) showed altered mechanical properties in response to *in vitro* fMLP treatment (Fig. 4B).

Since cell lines are only models of primary cells, we also measured and compared the compliance of primary human hematopoietic stem cells (CD34+) with *in vitro* differentiated CD34+ cells as well as with primary human neutrophils and monocytes. Consistent with the cell line results, the primary human neutrophils and the CD34+-derived neutrophils were respectively 3-fold and 4-fold more compliant than primary CD34+ stem cells (Fig. 4C). The primary monocytes were also significantly more compliant than primary stem cells (Fig. S7). Furthermore, macrophages derived from the differentiation of CD34+ stem cells were significantly (p<0.01) less compliant than undifferentiated CD34+ stem cells (Fig. S8). Of note, since monocytes turn into macrophages *in vivo*, we also measured primary monocytes before and after they had turned into macrophages in culture and found the latter to be 40% less compliant than the monocytes, consistent with all other results reported above. Thus, cell compliance is a lineage-specific characteristic material property of both cell lines and primary myeloid cells.

## Discussion

We have shown that differentiating myeloid precursor cells modulate their viscoelastic properties in a lineage-specific manner. This is in line with some previous studies on the modulation of mechanical properties during stem cell differentiation. Increase in deformability and reduction in viscosity were found when HL60 cells were induced to differentiate along the neutrophil lineage [27]. Beyond blood cells, distinct viscoelastic properties have been found in primary chondrocytes, osteoblasts and adipocytes on one hand and primary mesenchymal stem cells on the other, but no differentiation of the latter was carried out [11]. Recently, changes in mechanical properties similar to those reported for primary cells [11] were obtained during *in vitro* osteogenic and adipogenic differentiation of mesenchymal stem cells [10]. In general, the functional relevance of changes in viscoelastic properties of differentiating cells reported in these and similar studies remained speculative and unclear. A critical step in this regard was made when the regulatory role of cell compliance for long time migration was confirmed for neutrophils [5]. However, this could not be linked with lineage specificity since there were no other lineages to compare with. In this paper, we present what is to our knowledge, the first multilineage demonstration of fate specificity and functional relevance of the viscoelastic properties of differentiating stem/precursor cells.

Measuring the mechanical properties of whole single cells in a non-contact way is not trivial. Tactile contact with the measuring probe such as AFM tips can create artifacts due to cell adhesive properties [28]. Optical stretching is contact-free and assesses cell mechanics in suspension — mimicking the circulatory phase environment of monocytes and neutrophils. The integration of the optical stretcher with microfluidic delivery of cells allows rapid measurement of large numbers of cells, which in turn enables the expedient tracking of time-dependent processes such as differentiation by many repeated measurements on short, well-defined time points over the course of days. The fact that cells in suspension assume a near spherical shape has the added benefit that cell size can be determined and tracked very accurately (see Fig. S3), which is much more involved in an attached state. Moreover, mechanical phenotyping using the OS works on a single-cell level, which is important as cell response, for example to TNF-α, has recently been shown as a binary, stochastic process, which had previously been obscured in bulk measurements [29]. An apparent limitation of this method relates to macrophages, which have to be detached from a substrate for measurement in the suspended state. Yet, it is remarkable that we are able to make accurate predictions of cellular function in an attached state (migration in peripheral tissue) by analyzing the mechanical properties measured when these cells are in suspension. An interesting recent technological development in this regard is the use of hydrodynamic stretching for screening even larger cell populations in a short amount of time [30]. Mechanical properties have even been shown to be predictive for the differentiation potential of certain stem cells [31].

Of course, cell mechanics is rooted in the molecular make-up of the cell. Cell mechanical properties are determined by the cytoskeleton, an interconnected network of filamentous polymers and regulatory proteins, which links the membrane through the cytoplasm to the nucleus and gives the cell physical integrity [25]. Recent biochemical work has provided a catalogue of cytoskeletal proteins that are up- or down-regulated during the differentiation of HL60 cells into neutrophils, monocytes and macrophages [32]. It was found that undifferentiated cells, neutrophils and monocytes, but not macrophages, are deficient in nesprin-1 and -2 giant isoforms, SUN1, vimentin, plectin and lamins A/C while actin and SUN2 are present [32], [33]. How the material properties of biological cells derive from these cytoskeletal details is still largely unknown and a topic of intense research effort [1], [2]. Nevertheless, our findings demonstrate that the emergent mechanical properties of cells are robust and able to distinguish between lineages.

## Conclusions

We have demonstrated the lineage- and function-specificity of the viscoelastic properties of differentiating stem/precursor cells. The lineage-specificity enables monitoring stem cell differentiation by mechanical phenotyping. The function-specificity shows the importance of material properties for understanding cell biology. Our results indicate that cell mechanical properties constitute a defining feature of cell biological function and can be used as an inherent, sensitive marker for monitoring differentiation and as a novel therapeutic target to interfere with aberrant cell migration and circulation behaviour.

## Methods

### Ethics Statement

Ethical approval was obtained from the Cambridgeshire 2 Research Ethics Committee, and written informed consent, or written assent from next-of-kin where appropriate, were obtained in all cases in accordance with the Declaration of Helsinki.

### Cell culture and differentiation

We used HL-60/S4 cells [34], a rapidly differentiating clone or sub-line of HL-60 cells, as a model for myeloid differentiation. The cell line was a gift from Donald and Ada Olins of the Department of Pharmaceutical Sciences, College of Pharmacy, University of New England. Cells were cultured and induced to differentiate along the myeloid lineage following well-established protocols [33]. Differentiation was confirmed in various ways (see Methods S1 and Fig. S1). Primary human neutrophils and monocytes were isolated from peripheral blood taken from healthy donors at the Department of Medicine, Addenbrooke's Hospital, University of Cambridge. Human cord blood-derived CD34+ stem cells were obtained from Translational Research Laboratory, Department of Haematology, University of Cambridge. Details of cell culture and differentiation for both the cell line model and primary HSCs as well as the preparation of primary neutrophils and monocytes can be found in Methods S1.

### Optical stretching and determination of creep compliance

The principle, setup and handling of the optical stretcher have been described previously [5], [16], [35]. The forces that trap and deform the cell outwardly along the surface arise from the change in the refractive index at the cell-medium interface and the ensuing conservation of momentum [36]. The axial strain during optical stretching is given by
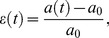
(4)where *a*_o_ is the semi-major axis of the unstretched cell and *a*(*t*) is the time-varying semi-major axis measured (Fig. 1B). The optical stress on the cells is computed using an electromagnetic wave model [37], which requires knowledge of the cells' average refractive index. We measured the refractive index of the cells during differentiation using a digital holographic microscope [38]. The strain is normalized by the peak value of the calculated optical stress *σ*_0_ and a geometric factor *F*_g_ to give the creep compliance.
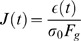
(5)The geometric factor is calculated as described elsewhere [18] to account for cell size and stress distribution. Of note, cell size changes characteristically upon differentiation (see Fig. S3). Thus, by this normalization the mechanical properties reported here are decoupled from the variation in the dielectric characteristics and in the size of cells during differentiation.

### Statistical analysis

For each cell type or treatment condition the number of cells per OS experiment was *n*≥30. Compliance data are presented as mean ± SEM. Representative compliance data were chosen from total number of independent experiments, *N*>5 for all cell lines and *N*≥3 for primary cells. Overall, the number of individual cells analyzed totaled about 5000. The rheological parameters *J*_o_ and *β* were confirmed in two independent experiments. For each cell type or treatment condition the number of cells per advection experiment was *n*>20. Advection times are representative of 3 independent experiments and are presented as mean ± SEM. Statistical comparisons for all experiments were done using one-way ANOVA in Origin (OriginLab Corporation, Northampton, MA, USA), to account for multiple groups. Within ANOVA, significant differences were reported only where at least three different means comparison tests (Tukey, Bonferroni and Dunn-Sidak) simultaneously showed such differences. All box plots include whiskers at 5–95% range, horizontal box lines at 25–75% range, median line and the mean (inset box). In the parametric fitting 95% confidence intervals were computed, and extracted parameters were considered significant where confidence intervals did not include 0. All curve fitting was performed using Matlab (Mathworks, Natwick, MA).

## Supporting Information

Methods S1 (DOCX)

Figure S1 **Nuclear morphology as further proof of differentiation and cell viability during differentiation.** **A**. Representative confocal images (single slices) of cells stained with a green fluorescent cytoplasmic dye (Mitotracker Orange) and a red fluorescent nuclear dye (Syto 61). Neutrophils have lobulated nuclei (bands and segmented), usually 3–5 lobes but all lobes are not always visible in a 2D image. Monocytes have bean-shaped or ‘horse-shoe’-like nuclei and macrophages have bean-shaped or round nuclei. The undifferentiated cells have large round or large rough-edged nuclei. **B**. Neutrophils derived from the differentiation of primary human CD34+ stem cells also have lobulated nuclei. **C**. Cell viability during differentiation of HL60 cells. All macrophages were obtained at 24 h after induction, hence the low viability at 72 h after. **D**. Cell viability during differentiation of CD34+ SCs. Viability was low on day 1 (71%) but rose to about 99% from days 12 to 15 when neutrophils were present. However, Annexin V (green) and Propidium Iodide (red) stains indicate 10% apoptosis, as expected due to the presence of short-lived neutrophils. All scale bars in **A**, **B** and **D** are 10 µm.
(TIF)

Figure S2 **Distribution of creep compliance and stress- as well as set-up independence of lineage specificity in creep compliance.** **A**. The histograms show the distribution of peak compliance (at *t* = 4 s) for the HL60 and all differentiated lineages plotted in Fig. 1C. Although the populations overlap there are significant shifts in compliance (decrease to the left for macrophages and increase to the right for monocytes and neutrophils). **B**. Lineage specific differences in creep compliance measured at a higher power of 0.9 W per fibre almost identical to differences obtained at 0.7 W per fibre (Fig. 1C) illustrating the stress-independence of lineage specificity in creep compliance. For HL60, *n* = 62, neutrophils (Neu), *n* = 70, monocytes (Mono), *n* = 53 and macrophages (Mac), *n* = 41. **C**. Lineage-specificity in compliance is also reproduced in an OS set-up with a different geometry. Here, HL60, *n* = 40, neutrophils, *n* = 37, monocytes, *n* = 37 and macrophages, *n* = 36. It is quite impressive how few cells need to be measured to be able to distinguish the various lineages based on creep compliance. **D**. HL60 cells in the logarithmic growth phase (*n* = 29, 36, 40, from 0 h, respectively) have highly reproducible strains and this phase lasts about 24 h after which plateau is reached and differentiation experiments are not usually successful. **E**. There is no detectable drift in mechanical properties of logarithmic-phase HL60 cells after 8 weeks of routine maintenance in suspension culture (*n* = 34, 27, from 0 h, respectively). **F**. Peak strains (at *t* = 4 s) for strain experiments shown in **D** and **E**.
(TIF)

Figure S3 **Size changes during the differentiation of cells over several days.** Macrophages become larger with differentiation, neutrophils and monocytes become smaller. The cell radii shown (mean ± SEM) were obtained during the OS measurements reported in Fig. 2 (with the same number of cells in each measurement) and used for the proper normalization of the strain data to yield the creep compliance.
(TIF)

Figure S4 **Conversion of compliance to complex modulus using power law and mechanical models within quasi-linear viscoelastic regimes.** **A**. Two approximately linear viscoelastic regimes for cell line model: the first ending around 0.97 Pa, corresponds to about 0.75 W per fibre, thus, engendering the experiments at 0.7 W per fibre (all experiments reported in the main text). **B**. Complex modulus based on power law model. **C**. Complex modulus based on a mechanical model, here, Burgers' model, showing a transition in viscoelasticity at very high frequency, which implies the presence of distinct time scales cellular viscoelastic response to stress.
(TIF)

Figure S5 **Advection times at 10 mbar.** HL60 cells (*n* = 117) required a significantly longer (*p*<0.001) advection time of 0.43±0.07 s to go through the 10×12 µm channel compared to 0.13±0.03 s for monocytes (*n* = 88) and 0.15±0.04 s neutrophils (*n* = 49).
(TIF)

Figure S6 **F-actin depolymerization reduces transition times in all cell types.** The transition time dropped to less than 0.8 s in all cell types treated with 2 µM cytochalasin D (cytoD).
(TIF)

Figure S7 **Primary stem cells (CD34+) are less compliant than primary monocytes and have highly reproducible viscoelastic properties across different healthy donors.** **A**. CD34+SCs are less compliant than primary monocytes. **B**. Box plots for the results of **A**, showing highly significant difference (*p*<0.0001). **C**. Primary CD34+ stem cells from different donors show highly reproducible strains in OS measurements at 0.9 W per fibre. As in Fig. 2D and E, few cells were required to obtain reproducible results (for donors 1, 2 and 3, *n* = 27, 45, 36, respectively), a fact that is not trivial for diagnostic applications, considering that stem cells are very rare. Also, this near absence of variation in compliance of CD34+SCs from healthy donors suggests that OS is probably capable of discerning dysfunctional states in human populations.
(TIF)

Figure S8 **Macrophages derived from primary stem cells (CD34+) are less compliant than undifferentiated CD34+ stem cells.** **A**. Upon differentiation, CD34+ derived macrophages (CD34+ pMac, *n* = 32) become less compliant than undifferentiated CD34+SCs (*n* = 45). **B**. Box plots for the results of **A**, showing significant difference (*p*<0.01).
(TIF)

Video S1 **Short time scale advection of macrophages through microfluidic channels.** Macrophages obviously need quite some time to deform before they can slip through. Video presented in real time.
(WMV)

Video S2 **Short time scale advection of monocytes through microfluidic channels.** Monocytes need much less time than the macrophages in Video S1 to slip through the same channels and at the same driving pressure. Video presented in real time.
(WMV)

## References

[1] Kasza KE, Rowat AC, Liu J, Angelini TE, Brangwynne CP, et al (2007) The cell as a material. Curr Opin Cell Biol 19: 101–107 doi:10.1016/j.ceb.2006.12.002. 17174543 10.1016/j.ceb.2006.12.002

[2] Trepat X, Deng L, An SS, Navajas D, Tschumperlin DJ, et al (2007) Universal physical responses to stretch in the living cell. Nature 447: 592–595 doi:10.1038/nature05824. 17538621 10.1038/nature05824 PMC2440511

[3] Remmerbach TW, Wottawah F, Dietrich J, Lincoln B, Wittekind C, et al (2009) Oral cancer diagnosis by mechanical phenotyping. Cancer Res 69: 1728–1732 doi:10.1158/0008-5472.CAN-08-4073. 19223529 10.1158/0008-5472.CAN-08-4073

[4] Chowdhury F, Na S, Li D, Poh Y-C, Tanaka TS, et al (2010) Material properties of the cell dictate stress-induced spreading and differentiation in embryonic stem cells. Nat Mater 9: 82–88 doi:10.1038/nmat2563. 19838182 10.1038/nmat2563 PMC2833279

[5] Lautenschläger F, Paschke S, Schinkinger S, Bruel A, Beil M, et al (2009) The regulatory role of cell mechanics for migration of differentiating myeloid cells. Proc Natl Acad Sci USA 106: 15696–15701 doi:10.1073/pnas.0811261106. 19717452 10.1073/pnas.0811261106 PMC2747182

[6] Tsai MA, Waugh RE, Keng PC (1996) Cell cycle-dependence of HL-60 cell deformability. Biophys J 70: 2023–2029 doi:10.1016/S0006-3495(96)79768-0. 8785361 10.1016/S0006-3495(96)79768-0 PMC1225171

[7] Pelling AE, Veraitch FS, Chu CPK, Mason C, Horton MA (2009) Mechanical dynamics of single cells during early apoptosis. Cell Motil Cytoskeleton 66: 409–422 doi:10.1002/cm.20391. 19492400 10.1002/cm.20391

[8] Tamayo P, Slonim D, Mesirov J, Zhu Q, Kitareewan S, et al (1999) Interpreting patterns of gene expression with self-organizing maps: methods and application to hematopoietic differentiation. Proc Natl Acad Sci USA 96: 2907–2912. 10077610 10.1073/pnas.96.6.2907 PMC15868

[9] Jain KK (2010) The Handbook of Biomarkers. 1st ed. New York: Springer. p.

[10] Yu H, Tay CY, Leong WS, Tan SCW, Liao K, et al (2010) Mechanical behavior of human mesenchymal stem cells during adipogenic and osteogenic differentiation. Biochem Biophys Res Commun 393: 150–155 doi:10.1016/j.bbrc.2010.01.107. 20117089 10.1016/j.bbrc.2010.01.107

[11] Darling EM, Topel M, Zauscher S, Vail TP, Guilak F (2008) Viscoelastic properties of human mesenchymally-derived stem cells and primary osteoblasts, chondrocytes, and adipocytes. J Biomech 41: 454–464 doi:10.1016/j.jbiomech.2007.06.019. 17825308 10.1016/j.jbiomech.2007.06.019 PMC2897251

[12] Collins SJJ (1987) The HL-60 promyelocytic leukemia cell line: proliferation, differentiation, and cellular oncogene expression. Blood 70: 1233–1244. 3311197

[13] Mercier FE, Ragu C, Scadden DT (2011) The bone marrow at the crossroads of blood and immunity. Nat Rev Immunol 12: 49–60 doi:10.1038/nri3132. 22193770 10.1038/nri3132 PMC4013788

[14] Cowburn AS, Condliffe AM, Farahi N, Summers C, Chilvers ER (2008) Advances in neutrophil biology: clinical implications. Chest 134: 606–612 doi:10.1378/chest.08-0422. 18779195 10.1378/chest.08-0422 PMC2827863

[15] Shi C, Pamer EG (2011) Monocyte recruitment during infection and inflammation. Nat Rev Immunol 11: 762–774 doi:10.1038/nri3070. 21984070 10.1038/nri3070 PMC3947780

[16] Guck J, Ananthakrishnan R, Mahmood H, Moon TJ, Cunningham CC, et al (2001) The optical stretcher: a novel laser tool to micromanipulate cells. Biophys J 81: 767–784 doi:10.1016/S0006-3495(01)75740-2. 11463624 10.1016/S0006-3495(01)75740-2 PMC1301552

[17] Guck J, Schinkinger S, Lincoln B, Wottawah F, Ebert S, et al (2005) Optical deformability as an inherent cell marker for testing malignant transformation and metastatic competence. Biophys J 88: 3689–3698 doi:10.1529/biophysj.104.045476. 15722433 10.1529/biophysj.104.045476 PMC1305515

[18] Wottawah F, Schinkinger S, Lincoln B, Ananthakrishnan R, Romeyke M, et al (2005) Optical rheology of biological cells. Phys Rev Lett 94: 1–4 doi:10.1103/PhysRevLett.94.098103. 10.1103/PhysRevLett.94.098103 15784006

[19] Doerschuk CM, Beyers N, Coxson HO, Wiggs B, Hogg JC (1993) Comparison of neutrophil and capillary diameters and their relation to neutrophil sequestration in the lung. J Appl Physiol 74: 3040–3045. 8366005 10.1152/jappl.1993.74.6.3040

[20] Pagliara S, Chimerel C, Langford R, Aarts DG, Keyser UF (2011) Parallel sub-micrometre channels with different dimensions for laser scattering detection. Lab Chip 11: 3365–3368 doi:10.1039/c1lc20399a. 21804971 10.1039/c1lc20399a

[21] Wakatsuki T, Schwab B, Thompson NC, Elson EL (2001) Effects of cytochalasin D and latrunculin B on mechanical properties of cells. J Cell Sci 114: 1025–1036. 11181185 10.1242/jcs.114.5.1025

[22] Goethem EV, Poincloux R, Gauffre F, Maridonneau-Parini I, Cabec VL, et al (2010) Matrix architecture dictates three-dimensional migration modes of human macrophages: differential involvement of proteases and podosome-like structures. J Immunol 184: 1049–1061 doi:10.4049/jimmunol.0902223. 20018633 10.4049/jimmunol.0902223

[23] Phillipson M, Kubes P (2011) The neutrophil in vascular inflammation. Nat Med 17: 1381–1390 doi:10.1038/nm.2514. 22064428 10.1038/nm.2514 PMC7095830

[24] Aldonyte R, Jansson L, Piitulainen E, Janciauskiene S (2003) Circulating monocytes from healthy individuals and COPD patients. Respir Res 4: 11 doi:10.1186/1465-9921-4-11. 14624669 10.1186/1465-9921-4-11 PMC239032

[25] Fletcher DA, Mullins RD (2010) Cell mechanics and the cytoskeleton. Nature 463: 485–492 doi:10.1038/nature08908. 20110992 10.1038/nature08908 PMC2851742

[26] Verghese MW, Kneisler TB, Boucheron JA (1996) P2U agonists induce chemotaxis and actin polymerization in human neutrophils and differentiated HL60 cells. J Biol Chem 271: 15597–15601. 8663069 10.1074/jbc.271.26.15597

[27] Hallows KR, Frank RS (1992) Changes in mechanical properties with DMSO-induced differentiation of HL-60 cells. Biorheology 29: 295–309. 1298447 10.3233/bir-1992-292-309

[28] Stewart MP, Toyoda Y, Hyman AA, Müller DJ (2012) Tracking mechanics and volume of globular cells with atomic force microscopy using a constant-height clamp. Nat Protoc 7: 143–154 doi:10.1038/nprot.2011.434. 22222789 10.1038/nprot.2011.434

[29] Tay S, Hughey JJ, Lee TK, Lipniacki T, Quake SR, et al (2010) Single-cell NF-kappaB dynamics reveal digital activation and analogue information processing. Nature 466: 267–271 doi:10.1038/nature09145. 20581820 10.1038/nature09145 PMC3105528

[30] Gossett DR, Tse HTK, Lee SA, Ying Y, Lindgren AG, et al (2012) Hydrodynamic stretching of single cells for large population mechanical phenotyping. Proc Natl Acad Sci USA 109: 7630–7635 doi:10.1073/pnas.1200107109. 22547795 10.1073/pnas.1200107109 PMC3356639

[31] González-Cruz RD, Fonseca VC, Darling EM (2012) Cellular mechanical properties reflect the differentiation potential of adipose-derived mesenchymal stem cells. Proc Natl Acad Sci USA 109: 1523–1529 doi:10.1073/pnas.1120349109. 10.1073/pnas.1120349109 PMC3386052 22615348

[32] Olins AL, Hoang TV, Zwerger M, Herrmann H, Zentgraf H, et al (2009) The LINC-less granulocyte nucleus. Eur J Cell Biol 88: 203–214 doi:10.1016/j.ejcb.2008.10.001. 19019491 10.1016/j.ejcb.2008.10.001 PMC2671807

[33] Olins AL, Buendia B, Herrmann H, Lichter P, Olins DE (1998) Retinoic Acid Induction of Nuclear Envelope-Limited Chromatin Sheets in HL-60. Exp Cell Res 104: 91–104. 10.1006/excr.1998.4210 9828104

[34] Leung M, Sokoloski JA, Sartorelli AC (1992) Changes in Microtubules, Microtubule-associated Proteins, and Intermediate Filaments during the Differentiation of HL-60 Leukemia Cells. Cancer Res 52: 949–954. 1737356

[35] Lincoln B, Schinkinger S, Travis K, Wottawah F, Ebert S, et al (2007) Reconfigurable microfluidic integration of a dual-beam laser trap with biomedical applications. Biomed Microdevices 9: 703–710 doi:10.1007/s10544-007-9079-x. 17505883 10.1007/s10544-007-9079-x

[36] Guck J, Ananthakrishnan R, Moon TJ, Cunningham CC, Käs J (2000) Optical deformability of soft biological dielectrics. Phys Rev Lett 84: 5451–5454. 10990966 10.1103/PhysRevLett.84.5451

[37] Boyde L, Chalut KJ, Guck J (2009) Interaction of Gaussian beam with near-spherical particle: an analytic-numerical approach for assessing scattering and stresses. J Opt Soc Am A Opt Image Sci Vis 26: 1814–1826. 19649116 10.1364/josaa.26.001814

[38] Chalut KJ, Ekpenyong AE, Clegg WL, Melhuish IC, Guck J (2012) Quantifying cellular differentiation by physical phenotype using digital holographic microscopy. Integr Biol (Camb) 4: 280–284 doi:10.1039/c2ib00129b. 22262315 10.1039/c2ib00129b

