# Supplementary material for: Viscoelastic Properties of Differentiating Blood Cells Are Fate- and Function-Dependent
Source: PLoS One. 2012 Sep 27;7(9):e45237. doi: 10.1371/journal.pone.0045237 (PMC3459925; doi:10.1371/journal.pone.0045237)
Supplement: Methods S1 — (DOCX) [file pone.0045237.s001.docx]

**SUPPORTING INFORMATION**

**Viscoelastic Properties of Differentiating Blood Cells are**

**Fate- and Function-Dependent**

**Andrew E. Ekpenyong^1^**, **Graeme Whyte^1^, Kevin Chalut^1^, Stefano Pagliara^1^, Franziska Lautenschläger^1^, Christine Fiddler^2^, Stephan Paschke^3^, Ulrich F. Keyser^1^, Edwin R. Chilvers^2^ and Jochen Guck^1,4*^**

^1^Cavendish Laboratory, Department of Physics, University of Cambridge, CB3 0HE, UK

^2^Department of Medicine, University of Cambridge School of Clinical Medicine, Addenbrooke’s and Papworth Hospitals, Cambridge CB2 0QQ, UK

^3^Department of Surgery, University of Ulm, 89075 Ulm, Germany

^4^ Biotechnology Center, Technische Universität Dresden, 01307 Dresden, Germany

****To whom correspondence should be addressed.

Prof. Dr. Jochen Guck

Biotechnology Centre, Technische Universität Dresden

Tatzberg 47/49, 03107 Dresden, Germany

Email: [jochen.guck@biotec.tu-dresden.de](mailto:jochen.guck@biotec.tu-dresden.de)

Phone: +49 351 463 40330

**Supporting Methods S1**

**Ethics Statement**

Ethical approval was obtained from the Cambridgeshire 2 Research Ethics Committee, and written informed consent, or written assent from next-of-kin where appropriate, were obtained in all cases in accordance with the Declaration of Helsinki.

**Cell culture and differentiation**

The rapidly differentiating clone or subline of HL60 cells, called HL-60/S4 [1], which we used for the model differentiation experiments, was a gift from Donald E. Olins and Ada L. Olins. Cells were cultured and induced to differentiate along the myeloid lineage following well established protocols [2–4]. In brief, HL-60/S4 cells were cultivated in RPMI 1640 medium supplemented with 2 mM L-Glutamine (Sigma R8758), 10% heat inactivated fetal bovine serum (FBS, Sigma F9665) and 1% penicillin/streptomycin (Invitrogen P4333). Cultures were propagated at 37°C under 5% CO_2_ in 95% air, inside a humid incubator. Only cells in the logarithmic phase of growth were used for induction of differentiation. In this phase, viability was routinely above 98.5%, as determined using Trypan Blue dye (Invitrogen 15250-061). During induced differentiation all T-25 and T-75 flasks used for differentiation were covered with aluminum foil to protect against light which would alter the concentrations. The differentiation procedures for each cell type are tabulated below.

| **Cell type** | **Differentiation** |
| --- | --- |
| **HL60-derived**  **neutrophils (Neu)** | All-trans retinoic acid (ATRA or RA), C_20_H_28_O_2_, (Sigma R2625) was used for generating neutrophils. RA from stock solution was added to 1 µM. Cell density was rigorously kept at 1.5 x 10^5^ cells/ml after we found that the percentage of differentiated cells depended on cell density for all lineages. |
| **HL60-derived monocytes (Mono)** | 1-alpha, 25 Dihydroxyvitamin D3 (D3), 1,25(OH)_2_D3, (Sigma D1530) at 100 nM was used for inducing differentiation into monocytic cells. Cell density was rigorously kept at 1.5 x 10^5^ cells/ml. |
| **HL60-derived macrophages (Mac)** | Phorbol 12-myristate-13-acetate (TPA or PMA), C_36_H_56_O_8_, (Sigma, 79346) at a final concentration of 16 nM was used in generating macrophages. Cell density was rigorously kept at 2.5 x 10^5^ cells/ml. |

**Preparation of primary human neutrophils and monocytes**

Neutrophils and monocytes were isolated from the blood of healthy adult volunteers using dextran sedimentation, followed by centrifugation through discontinuous plasma Percoll™ gradients [5]. Purified neutrophils, derived from the 42%/51% Percoll™ interface, were harvested, washed and re-suspended at 5 x 10^6^ cells/ml in PBS with CaCl_2_ and MgCl_2_ (Sigma D8662). Neutrophils prepared in this manner were routinely >95% pure, as assessed by examining methanol fixed and REASTAIN Quick-Diff stained cytospin preparations, >99% viable, as assessed by Trypan blue exclusion, unprimed with respect to basal and fMLP-stimulated superoxide anion release and if maintained in plasma circulate in an entirely physiological manner if re-injected *in vivo* [6]. Peripheral blood mononuclear cells were collected from the upper platelet-poor plasma/42% Percoll™ interface. Monocytes were subsequently purified (up to 80%) by plating PBMCs overnight in culture flasks and removing any unbound cells.

**Preparation of primary human cord blood stem cells**

Human cord blood-derived CD34+ stem cells are routinely extracted at the Translational Research Laboratory, Department of Haematology, University of Cambridge, and made available to accredited Cambridge University researchers at service cost. From this laboratory we purchased fresh (same day) CD34+ stem cells extracted from the umbilical cords and placentae of healthy newborns and purified by standard immuno-magnetic sorting and FACS.

**Differentiation of primary human cord blood stem cells**

The primary human cord blood-derived CD34+ SCs were cultured and differentiated into neutrophils using a two-stage protocol [7]. The first stage consisted in the induction of CD34+ SCs to differentiate towards early promyelocytic cells by *ex vivo* suspension culture in Iscove’s Modified Dulbecco’s Medium, (IMDM, Sigma I6529), supplemented with 10% heat-denatured FBS (Sigma F9665), 1% penicillin/streptomycin (Invitrogen P4333), 100 ng/ml human stem cell factor, SCF (Sigma S7901), and 100 ng/ml interleukin-3 (IL-3, Sigma I1646) for three days. The second stage involved the transfer of promyelocytes into a medium consisting of IMDM, 10% FBS, 100 ng/ml of SCF, IL-3 and granulocyte colony-stimulating factor, (G-CSF, Sigma G0407) leading to their terminal differentiation into mature neutrophils from the 10^th^ day of culture. OS measurements and confocal microscopy verification of nuclear morphology were made simultaneously on the differentiated cells on days 10, 11, 12, 13, 14 and 15 (Fig. S1). Cell viability and apoptosis were checked using Trypan blue dye and Annexin V/propidium iodide (ApoDETECT Annexin V-FITC Kit, Invitrogen, 331200). The manufacturer’s protocol was used for the apoptosis detection. Although viability was over 97% from day 11 to day15, about 10% of cells were apoptotic on day 15 (Fig. S1D) as expected due to short-lived neutrophils.

For differentiation into monocytes and subsequently macrophages, the same two-stage protocol was used, except that in stage two, GM-CSF was not added, ensuring that monocytes, neutrophils and macrophages were obtained [8]. From day 15 to day 22, only attached cells were maintained in culture, which were identified as macrophages.

**Further proofs of differentiation**

In addition to functional assays (Fig. 4B) to test for successful differentiation of cells and the obvious phenotypic changes accompanying differentiation (adherence to substrate for macrophages, near-cessation of proliferation for neutrophils, reduction in size for monocytes and neutrophils) into mature myeloid cells, we carried out two more phenotypic assays: characterization of nuclear shape by double-staining confocal microscopy and re-induction of differentiation. For determining nuclear morphology (Fig. S1) and nuclear to cytoplasmic ratio, live cells were stained with two dyes: one for nuclei (SYTO 61, Invitrogen 775304) and the other for the cytoplasm including mitochondria (MitoTracker Orange CMTMRos, Invitrogen, 815301). After incubation in culture media with the two dyes each at a final concentration of 10 µM for 30-45 minutes, cells were centrifuged, resuspended in culture media, incubated for 10 mins, washed again in culture media, placed on a depression slide and brought to a Leica SP5 inverted confocal microscope where confocal images were taken using a 63x oil-immersion objective. We developed an in-house code in Matlab (Mathworks, Natwick, MA), for simultaneous segmentation of the nucleus and the cytoplasm in cross-sectional images of single cells. Both HL60-derived neutrophils and CD34+-derived neutrophils showed nuclear lobulation (3-5 lobes appearing as either as segmented lobes or banded lobes) characteristic of primary neutrophils (Fig.S1A, B). Monocytes showed typical horse-shoe-shaped nuclei. Since over 98% of macrophages become attached to culture flask and display pseudopodia-like protrusions, their successful differentiation was obvious without any nuclear morphologic characterization though their low nuclear to cytoplasmic ratio (about 35%) and somewhat ‘horse-shoe’-like nuclei (Fig. S1) corroborate with their status as mature monocytes. Furthermore, addition of 16 nM PMA to HL60-derived neutrophils resulted in massive necrosis within 12 h while the same treatment on HL60-derived monocytes yielded macrophages in 24 h.

**Further rheological analysis**

The computed peak stress on the major axis of deformation was found to be linearly proportional to the input laser power thereby providing a direct correlation between the input laser functions and the calculated input stress. By using step functions for the input laser powers, the peak stresses were also step-functions. Classically, rheological experiments using step stress functions enable the measurement of creep compliance using the strain output. For viscoelastic materials in general, linear and non-linear regimes can be established by performing several creep tests and then plotting the isochronal curves for such materials [9]. The isochronal curves we plotted for HL60 cells showed at least two distinct viscoelastic regimes (Fig. S4A), indicating an overall non-linear viscoelastic response of HL60 cells. It should be noted that several previous studies using the optical stretcher involved the establishment of a linear or quasi-linear regime for measurements [10–12].

Since the first regime (from 0.2 W per fibre to 0.7 W per fibre or 0.55 Pa to 0.98 Pa) is approximately linear, all creep experiments on HL60 cells and the differentiated derivatives reported in the main text were performed at 0.7 W per fibre (1.4 W total power), to stay within this quasi-linear regime, which enables the conversion of creep compliance to complex modulus (storage and loss moduli) as well as from time to frequency domain. However, we found that lineage specific differences in creep compliance measured at 0.7 W per fibre were reproduced at 0.9 W per fibre (Fig. S2B), even though 0.9 W or 1.25 Pa is within a different regime compared to 0.7 W per fibre or 0.98 Pa (Fig. S4A).

We used power law models and mechanical models to fit the data and carried out several comparisons based on model independent algorithms [13] for converting compliance data to storage and loss moduli (Fig. S4B,C). For very high frequencies, power law models offer divergent insights from those of analog mechanical models. Basically, mechanical models predict distinct time scales in the viscoelastic response while power law models do not. For instance, there is an intersection between the loss modulus and the storage modulus (Fig. S4C) in the mechanical model, showing a transition. Note also the discrepancies between the two on time scales shorter than 0.1 s (Fig. 2A) which cannot be resolved unambiguously due to experimental limitations and we have extracted viscoelastic parameters where both are overlap.

While only the overlapping insights from both power law and mechanical models impinge upon the conclusions of this work, it might be interesting as future work, to explore the nature of cell viscoelasticity at very high frequencies or very short time scales (<0.1 s).

**Short time scale advection of cells**

The microfabrication of the fluidic chip to mimic the short time scale advection of cells relies on multilevel photolithography and replica molding. For the fabrication of the mold, a layer of SU-8 2005 (Microchem, Erlenbach, Switzerland) was deposited via spin coating (1000 rpm for 30 s) on a silicon wafer and exposed to UV light (1 s, 365-405 nm, 52 mW/cm^2^) through a quartz mask (Photodata Ltd, Hitchin, UK) patterned with an array of wires with two different widths, 10 µm and 12 µm. After development, a layer of SU-8 2015 (Microchem) was deposited by spin coating (2000 rpm for 30 s). A second mask patterned with two symmetrical reservoir chambers separated by a 50 µm gap and ending with two 1 mm-side circular pads was aligned relatively to the sample through a MJB4 mask aligner (Karl Suss, Garching, Germany) in a way that positioned the central region of the wire under the 50 µm-gap on the mask. The sample was exposed for 4 s and developed. The thickness of the obtained wires was 8 µm while the one of the reservoir was 25 µm as measured by a Dektak stylus profilometer (Veeco, Plainview, NY). Replica molding of the device, bonding, external connections to a computerized pressure-based flow control system and imaging were carried out as previously reported [14]. The length of the narrow channels was 50 *μ*m, chosen to enable the evaluation of advection times from entry point to a distance comparable to *in vivo* capillary constrictions as well as the role of cell adhesiveness in the process. Cells were advected sequentially through the microchannels (see Video S1) using physiological pressures achieved by computer controlled pumps. It is notable that the macrophages spent 94% or 3.74 ± 1.76 s of their advection (3.98 ± 1.77 s) time with slowly deforming into the entrance and first 15 *µ*m of the channel length. Once their cross-section became comparable to that of the channel, they simply slipped through the remainder at high speed, similar to the other cells. (VideoS1). This behavior of macrophages is consistent with a stiff object at short time scales and unambiguously eliminates cell adhesion as a limiting factor in cell advection through these narrow constrictions. The advection times of HL60 precursors, neutrophils and monocytes through the same channel at this pressure were all approximately an order of magnitude lower.

**Long timescale migration assay**

Boyden chambers were assembled using 5- and 12 µm pore Transwell filters (Costar)[15]. Human fibronectin (5 µg/ml) was used in coating both sides of the polycarbonate membrane. Cells were placed in the upper chamber while the lower chamber contained the chemoattractant fMLP (100 nM). Cells were left to migrate into the lower chamber for 3 h at 37°C, 5% CO_2_ and then counted under a microscope objective.

**Pharmacological assay and primary cells**

Since caution is required in interpreting model-dependent parameters such as the transition times obtained from mechanical models, we checked to see if the transition times were dependent on the actin cytoskeleton, a well-known major origin of cellular viscoelastic properties, by depolymerizing F-actin using Cytochalasin D. The transition time dropped to less than 0.8 s in all cell types treated with 2 µM Cytochalasin D (Supporting Fig. S6).

**Supporting References**

1. Leung M, Sokoloski JA, Sartorelli AC (1992) Changes in Microtubules, Microtubule-associated Proteins, and Intermediate Filaments during the Differentiation of HL-60 Leukemia Cells. Cancer Res 52: 949–954.

2. Bar-Shavit Z, Teitelbaum SL, Reitsma P, Hall A, Pegg LE, et al. (1983) Induction of monocytic differentiation and bone resorption by 1,25-dihydroxyvitamin D3. Proc Natl Acad Sci USA 80: 5907–5911.

3. Olins AL, Buendia B, Herrmann H, Lichter P, Olins DE (1998) Retinoic Acid Induction of Nuclear Envelope-Limited Chromatin Sheets in HL-60. Exp Cell Res 104: 91–104.

4. Olins AL, Hoang TV, Zwerger M, Herrmann H, Zentgraf H, et al. (2009) The LINC-less granulocyte nucleus. Eur J Cell Biol 88: 203–214. doi:10.1016/j.ejcb.2008.10.001.

5. Haslett C, Guthrie LA, Kopaniak MM, Johnston RB, Henson PM (1985) Modulation of multiple neutrophil functions by preparative methods or trace concentrations of bacterial lipopolysaccharide. Am J Pathol 119: 101–110.

6. Condliffe AM, Kitchen E, Chilvers ER (1998) Neutrophil priming: pathophysiological consequences and underlying mechanisms. Clin Sci (Lond) 94: 461–471.

7. Gaines P, Berliner N (2005) Differentiation and characterization of myeloid cells. Curr Protoc Immunol Chapter 22: Unit 22F.5. doi:10.1002/0471142735.im22f05s67.

8. Egeland T, Steen R, Quarsten H, Gaudernack G, Yang YC, et al. (1991) Myeloid differentiation of purified CD34+ cells after stimulation with recombinant human granulocyte-monocyte colony-stimulating factor (CSF), granulocyte-CSF, monocyte-CSF, and interleukin-3. Blood 78: 3192–3199.

9. Lakes RS (2004) Viscoelastic measurement techniques. Review of Scientific Instruments 75: 797. doi:10.1063/1.1651639.

10. Wottawah F, Schinkinger S, Lincoln B, Ananthakrishnan R, Romeyke M, et al. (2005) Optical rheology of biological cells. Phys Rev Lett 94: 1–4. doi:10.1103/PhysRevLett.94.098103.

11. Ekpenyong AE, Posey CL, Chaput JL, Burkart AK, Marquardt MM, et al. (2009) Determination of cell elasticity through hybrid ray optics and continuum mechanics modeling of cell deformation in the optical stretcher. Appl Opt 48: 6344–6354. doi:10.1364/AO.48.006344.

12. Remmerbach TW, Wottawah F, Dietrich J, Lincoln B, Wittekind C, et al. (2009) Oral cancer diagnosis by mechanical phenotyping. Cancer Res 69: 1728–1732. doi:10.1158/0008-5472.CAN-08-4073.

13. Evans R, Tassieri M, Auhl D, Waigh T (2009) Direct conversion of rheological compliance measurements into storage and loss moduli. Phys Rev E 80: 4–7. doi:10.1103/PhysRevE.80.012501.

14. Pagliara S, Chimerel C, Langford R, Aarts DG, Keyser UF (2011) Parallel sub-micrometre channels with different dimensions for laser scattering detection. Lab Chip 11: 3365–3368. doi:10.1039/c1lc20399a.

15. Lautenschläger F, Paschke S, Schinkinger S, Bruel A, Beil M, et al. (2009) The regulatory role of cell mechanics for migration of differentiating myeloid cells. Proc Natl Acad Sci USA 106: 15696–15701. doi:10.1073/pnas.0811261106.
