# Supplementary figures and images for: Viscoelastic Properties of Differentiating Blood Cells Are Fate- and Function-Dependent
Source: PLoS One. 2012 Sep 27;7(9):e45237. doi: 10.1371/journal.pone.0045237 (PMC3459925; doi:10.1371/journal.pone.0045237)

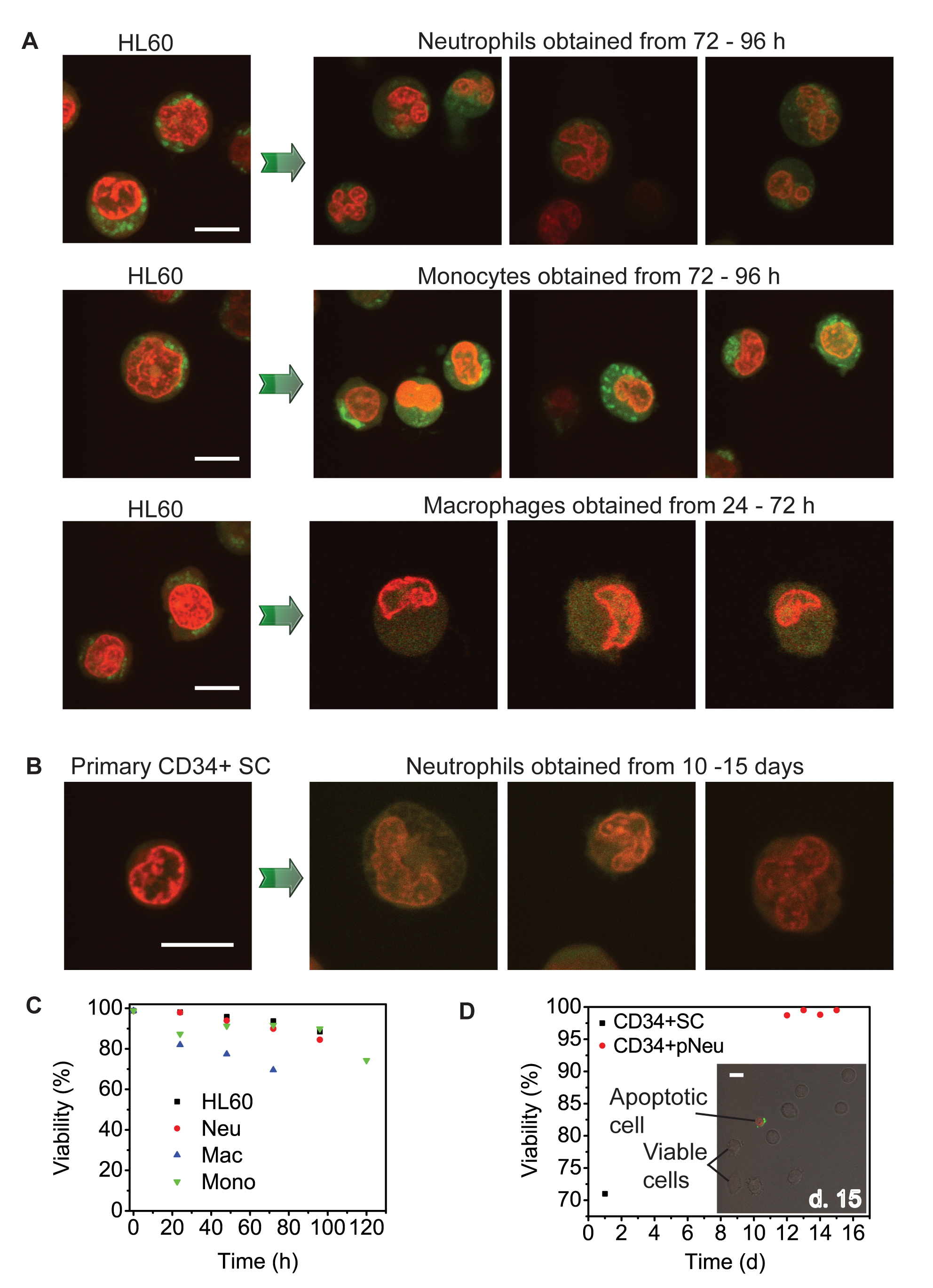

Supplement: Figure S1 — Nuclear morphology as further proof of differentiation and cell viability during differentiation. A. Representative confocal images (single slices) of cells stained with a green fluorescent cytoplasmic dye (Mitotracker Orange) and a red fluorescent nuclear dye (Syto 61). Neutrophils have lobulated nuclei (bands and segmented), usually 3–5 lobes but all lobes are not always visible in a 2D image. Monocytes have bean-shaped or ‘horse-shoe’-like nuclei and macrophages have bean-shaped or round nuclei. The undifferentiated cells have large round or large rough-edged nuclei. B. Neutrophils derived from the differentiation of primary human CD34+ stem cells also have lobulated nuclei. C. Cell viability during differentiation of HL60 cells. All macrophages were obtained at 24 h after induction, hence the low viability at 72 h after. D. Cell viability during differentiation of CD34+ SCs. Viability was low on day 1 (71%) but rose to about 99% from days 12 to 15 when neutrophils were present. However, Annexin V (green) and Propidium Iodide (red) stains indicate 10% apoptosis, as expected due to the presence of short-lived neutrophils. All scale bars in A, B and D are 10 µm. (TIF) [file pone.0045237.s002.tif]

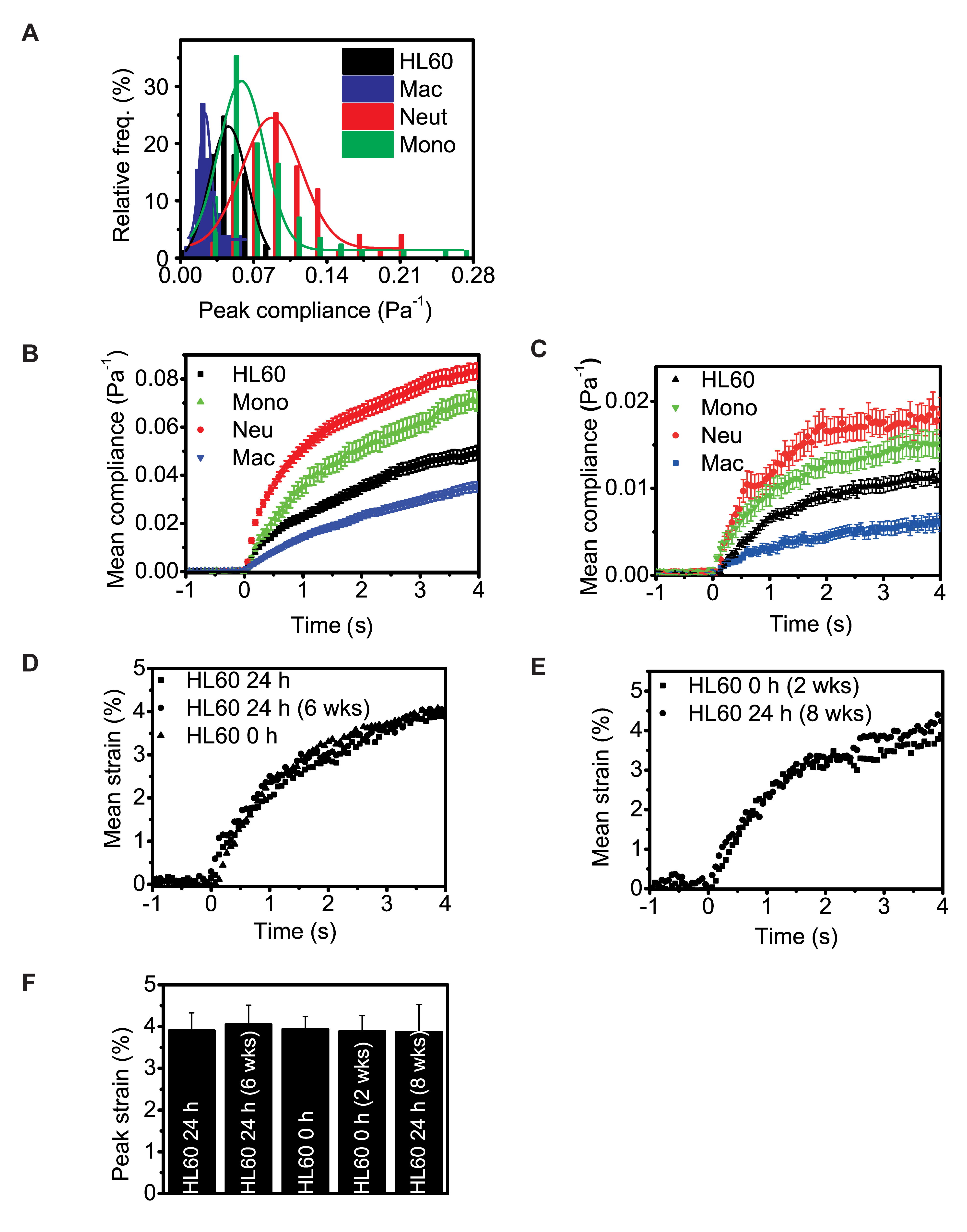

Supplement: Figure S2 — Distribution of creep compliance and stress- as well as set-up independence of lineage specificity in creep compliance. A. The histograms show the distribution of peak compliance (at t = 4 s) for the HL60 and all differentiated lineages plotted in Fig. 1C. Although the populations overlap there are significant shifts in compliance (decrease to the left for macrophages and increase to the right for monocytes and neutrophils). B. Lineage specific differences in creep compliance measured at a higher power of 0.9 W per fibre almost identical to differences obtained at 0.7 W per fibre (Fig. 1C) illustrating the stress-independence of lineage specificity in creep compliance. For HL60, n = 62, neutrophils (Neu), n = 70, monocytes (Mono), n = 53 and macrophages (Mac), n = 41. C. Lineage-specificity in compliance is also reproduced in an OS set-up with a different geometry. Here, HL60, n = 40, neutrophils, n = 37, monocytes, n = 37 and macrophages, n = 36. It is quite impressive how few cells need to be measured to be able to distinguish the various lineages based on creep compliance. D. HL60 cells in the logarithmic growth phase (n = 29, 36, 40, from 0 h, respectively) have highly reproducible strains and this phase lasts about 24 h after which plateau is reached and differentiation experiments are not usually successful. E. There is no detectable drift in mechanical properties of logarithmic-phase HL60 cells after 8 weeks of routine maintenance in suspension culture (n = 34, 27, from 0 h, respectively). F. Peak strains (at t = 4 s) for strain experiments shown in D and E. (TIF) [file pone.0045237.s003.tif]

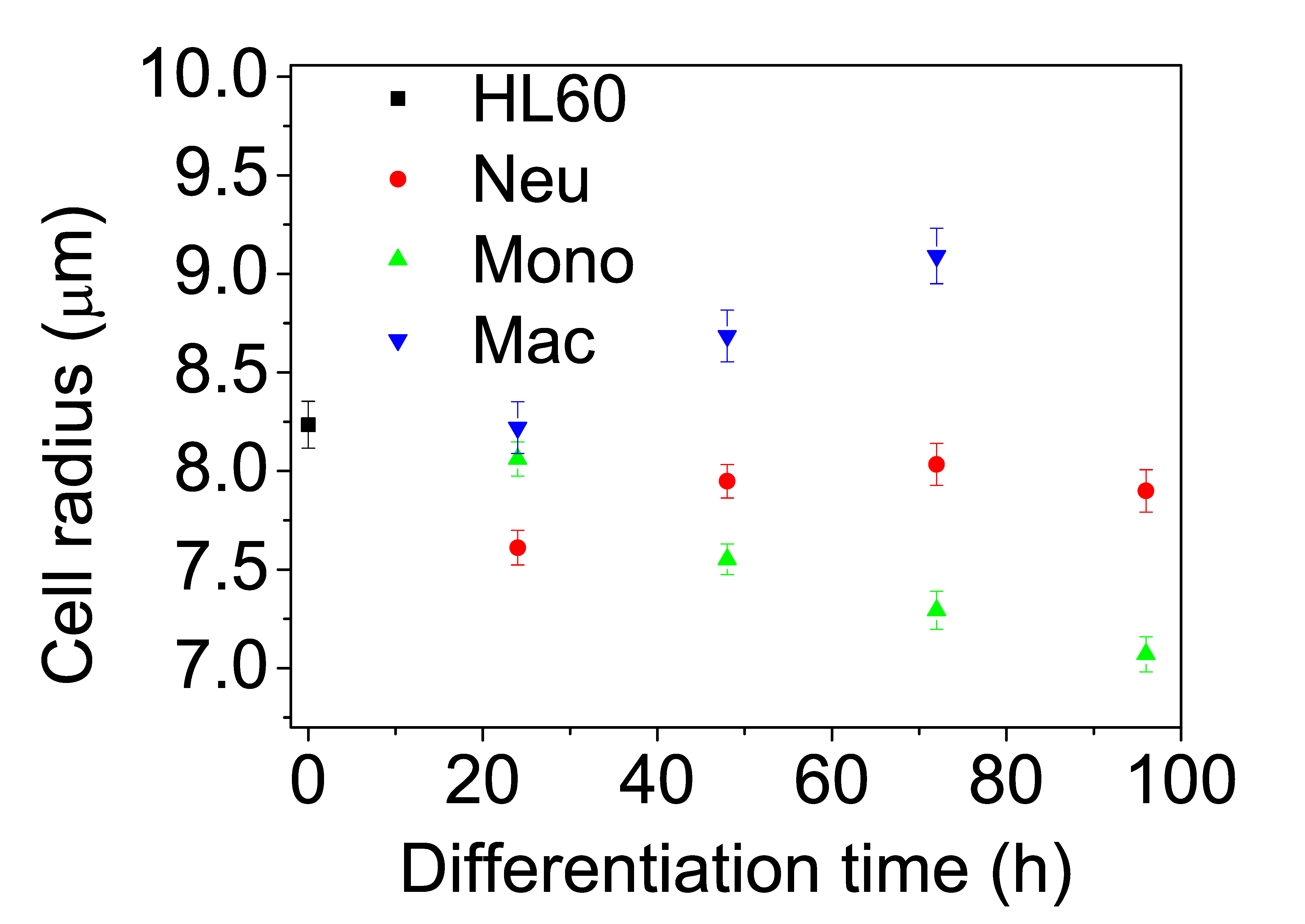

Supplement: Figure S3 — Size changes during the differentiation of cells over several days. Macrophages become larger with differentiation, neutrophils and monocytes become smaller. The cell radii shown (mean ± SEM) were obtained during the OS measurements reported in Fig. 2 (with the same number of cells in each measurement) and used for the proper normalization of the strain data to yield the creep compliance. (TIF) [file pone.0045237.s004.tif]

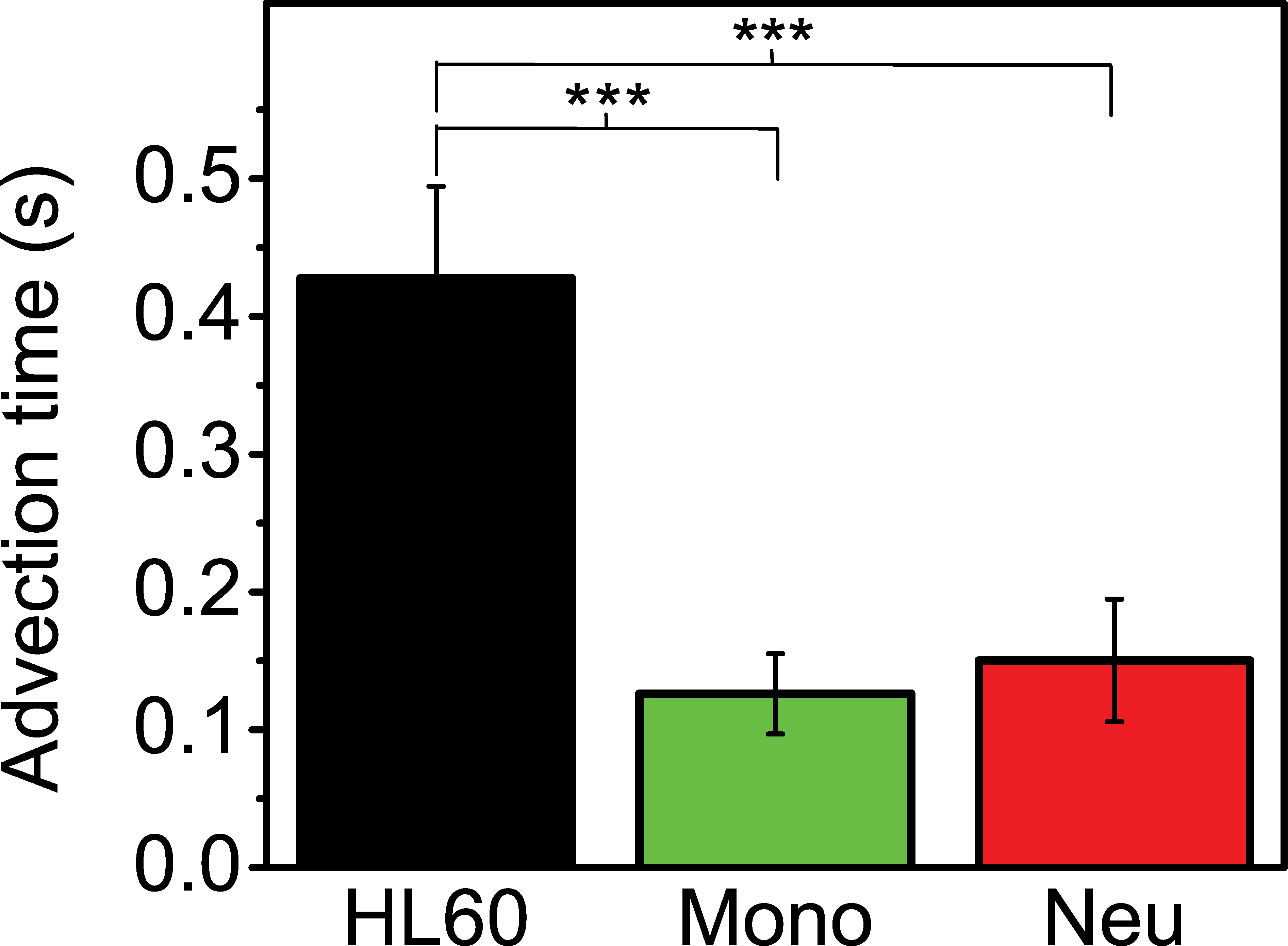

Supplement: Figure S5 — Advection times at 10 mbar. HL60 cells (n = 117) required a significantly longer (p<0.001) advection time of 0.43±0.07 s to go through the 10×12 µm channel compared to 0.13±0.03 s for monocytes (n = 88) and 0.15±0.04 s neutrophils (n = 49). (TIF) [file pone.0045237.s006.tif]

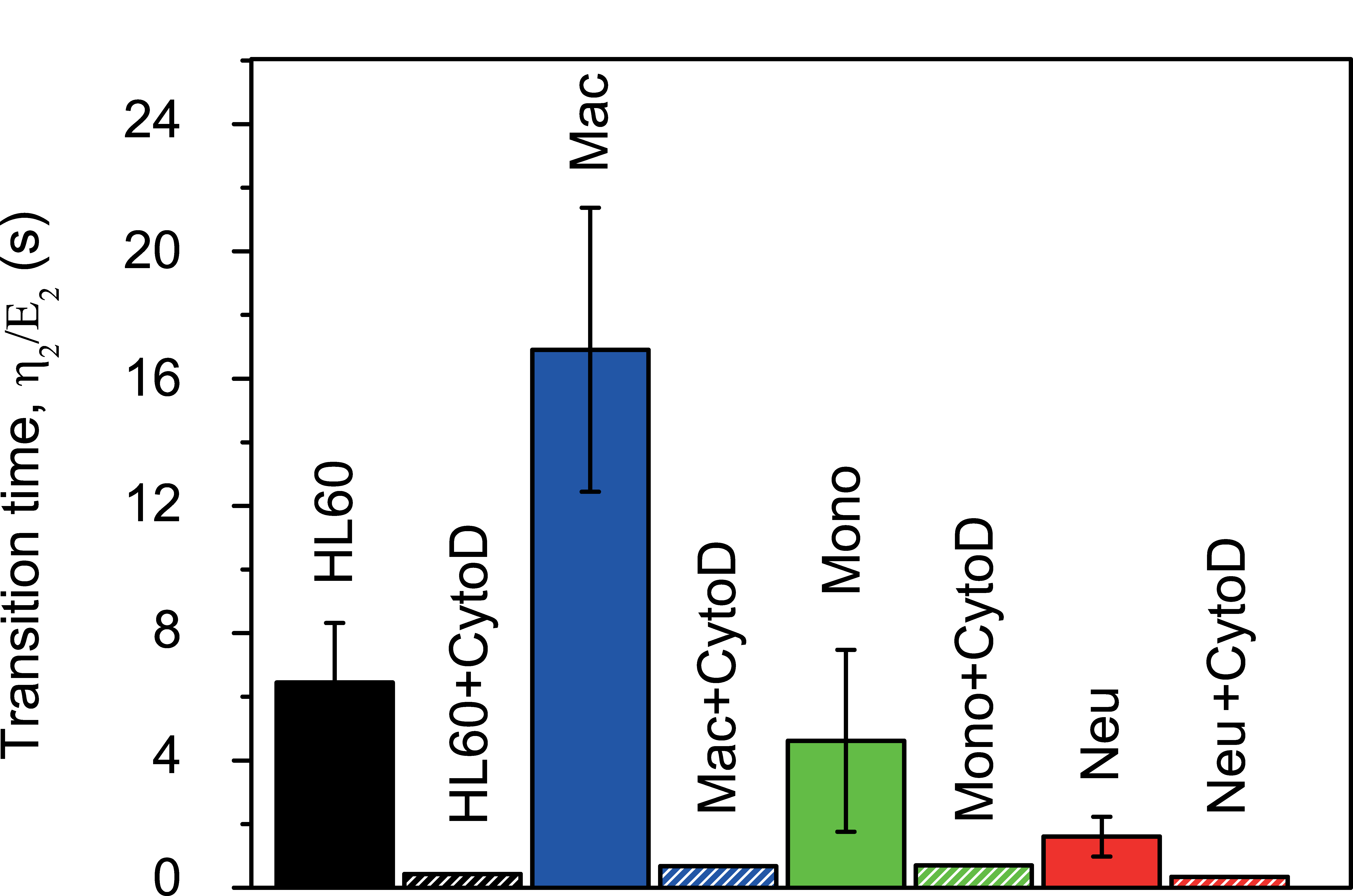

Supplement: Figure S6 — F-actin depolymerization reduces transition times in all cell types. The transition time dropped to less than 0.8 s in all cell types treated with 2 µM cytochalasin D (cytoD). (TIF) [file pone.0045237.s007.tif]

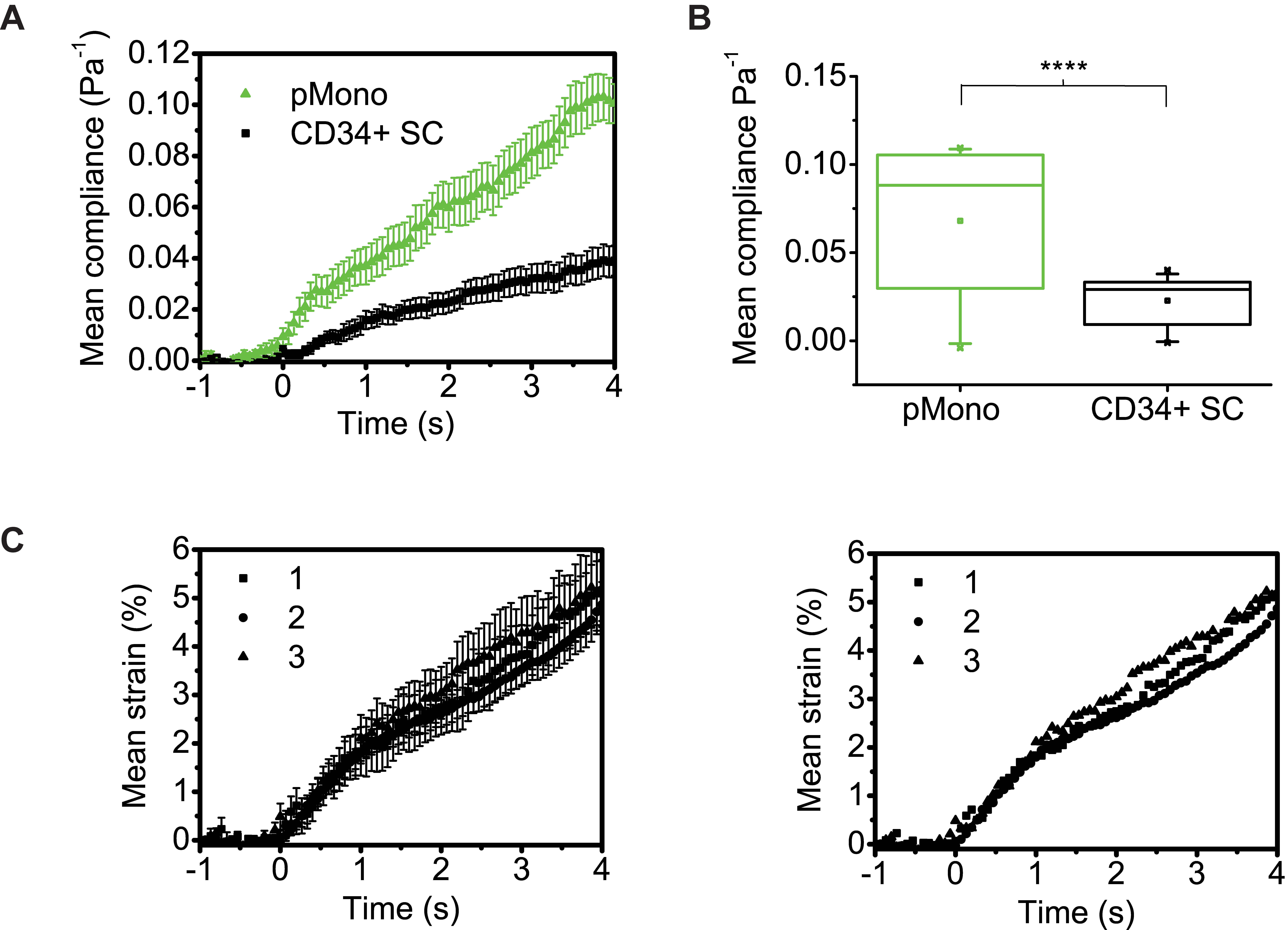

Supplement: Figure S7 — Primary stem cells (CD34+) are less compliant than primary monocytes and have highly reproducible viscoelastic properties across different healthy donors. A. CD34+SCs are less compliant than primary monocytes. B. Box plots for the results of A, showing highly significant difference (p<0.0001). C. Primary CD34+ stem cells from different donors show highly reproducible strains in OS measurements at 0.9 W per fibre. As in Fig. 2D and E, few cells were required to obtain reproducible results (for donors 1, 2 and 3, n = 27, 45, 36, respectively), a fact that is not trivial for diagnostic applications, considering that stem cells are very rare. Also, this near absence of variation in compliance of CD34+SCs from healthy donors suggests that OS is probably capable of discerning dysfunctional states in human populations. (TIF) [file pone.0045237.s008.tif]

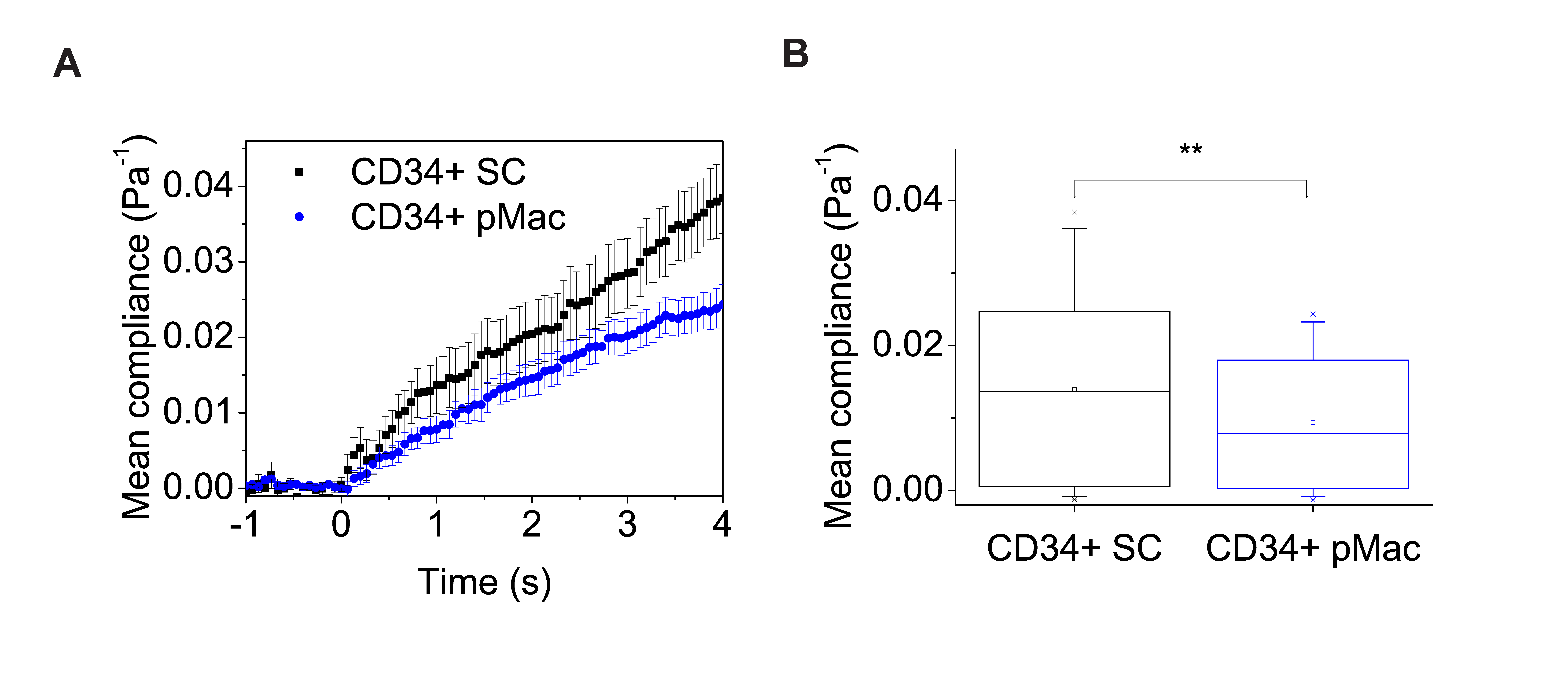

Supplement: Figure S8 — Macrophages derived from primary stem cells (CD34+) are less compliant than undifferentiated CD34+ stem cells. A. Upon differentiation, CD34+ derived macrophages (CD34+ pMac, n = 32) become less compliant than undifferentiated CD34+SCs (n = 45). B. Box plots for the results of A, showing significant difference (p<0.01). (TIF) [file pone.0045237.s009.tif]
